# Dihydrocapsaicin Enhances Tumor Necrosis Factor-α-Induced Apoptosis and G1 Cell Cycle Arrest in Human Cervical Cancer Cells Through TAK1-Mediated NF-κB and EGFR Pathways

**DOI:** 10.3390/ijms26115011

**Published:** 2025-05-22

**Authors:** Chantana Boonyarat, Hiroaki Sakurai, Yoshihiro Hayakawa, Suchada Chaiwiwatrakul, Rawiwun Kaewamatawong, Teeraporn Sadira Supapaan, Sureewan Duangjit, Benjabhorn Sethabouppha, Pornthip Waiwut

**Affiliations:** 1 Faculty of Pharmaceutical Sciences, Khon Kaen University, Khon Kaen 40002, Thailand; chaboo@kku.ac.th; 2 Department of Cancer Cell Biology, Graduate School of Medicine and Pharmaceutical Sciences, University of Toyama, Toyama 930-0194, Japan; hsakurai@pha.u-toyama.ac.jp; 3 Institute of Natural Medicine, University of Toyama, Toyama-shi, Toyama 930-0194, Japan; haya@inm.u-toyama.ac.jp; 4 Department of English, Faculty of Humanities and Social Sciences, Ubon Ratchathani Rajabhat University, Ubon Ratchathani 34000, Thailand; suchadachai65@gmail.com; 5 Faculty of Pharmaceutical Sciences, Ubon Ratchathani University, Ubon Ratchathani 34190, Thailand; rawiwun.k@ubu.ac.th (R.K.); teeraporn.s@ubu.ac.th (T.S.S.); sureewan.d@ubu.ac.th (S.D.); benjabhorn.s@ubu.ac.th (B.S.)

**Keywords:** dihydrocapsaicin, G1 cell cycle, apoptosis, TAK1, NF-κB, EGFR

## Abstract

Dihydrocapsaicin (DHC), a prominent capsaicinoid derived from red chili peppers, has shown cytotoxic effects against various cancer cell types. However, its role in modulating cytokine-induced survival and apoptotic signaling in cancer cells remains unclear. In this study, we investigated the effects of DHC on tumor necrosis factor-α (TNF-α)-induced cell cycle arrest and apoptosis in HeLa human cervical cancer cells. Our results demonstrate that DHC significantly enhances TNF-α-induced G1 phase cell cycle arrest and apoptosis by targeting the transforming growth factor-β-activated kinase 1 (TAK1)-mediated prosurvival pathways. DHC inhibited the phosphorylation of TAK1 and downstream effectors including IKKα, NF-κB p65, MAPKs (p38, JNK, ERK), Akt, and EGFR, thereby disrupting key signaling networks that typically confer resistance to TNF-α-induced cytotoxicity. Additionally, DHC suppressed the TNF-α-induced phosphorylation of EGFR at Ser-1046/1047 and Thr-669, sites critical for survival signaling. Co-treatment with DHC and TNF-α led to enhanced apoptotic features, including increased PARP-1 cleavage. These findings suggest that DHC sensitizes cervical cancer cells to cytokine-induced cell death by interfering with TAK1/NF-κB and EGFR signaling axes. Our study positions DHC as a promising candidate for combination therapies aimed at overcoming resistance in cancers with aberrant inflammatory and survival signaling.

## 1. Introduction

**Figure 1:**
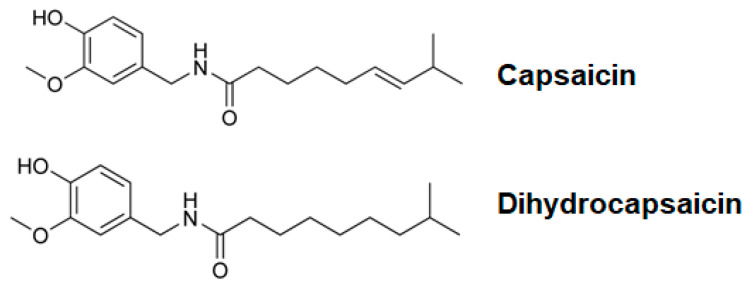
Structures of capsaicin and dihydrocapsaicin.

Capsicum species, commonly known as red peppers, are rich sources of bioactive compounds known as capsaicinoids, which are widely used not only as culinary additives but also as medicinal agents due to their diverse biological activities [1,2]. Capsaicinoids such as capsaicin and dihydrocapsaicin (DHC) (Figure 1) have demonstrated a range of pharmacological properties, including antioxidant, anti-inflammatory, antithrombotic, antiarthritic, and analgesic effects [3,4]. Additionally, they have been implicated in the regulation of lipid metabolism and glucose homeostasis, highlighting their potential for the prevention and treatment of chronic diseases [5]. Of particular interest is their selective cytotoxicity toward malignant cells while sparing normal, healthy cells, making them attractive candidates for cancer therapeutics [6].

Capsaicin has been shown to induce apoptosis and suppress proliferation across various human cancers, including pancreatic, lung, colon, bladder, and prostate malignancies, as well as urothelial carcinoma [7,8]. Epidemiological studies further suggest that the dietary intake of hot peppers may be inversely correlated with the incidence of lung and liver cancers [9]. Although the anti-cancer effects of capsaicin have been extensively investigated, DHC, which is chemically similar and equally abundant in peppers, remains comparatively understudied [10].

Emerging evidence indicates that DHC exerts anti-tumor effects through multiple mechanisms, including the inhibition of melanoma cell proliferation and metastasis via β-catenin signaling modulation, the induction of autophagy via the p53 pathway, and the activation of mitochondrial apoptosis through the generation of reactive oxygen species (ROS) and calcium flux in glioma cells [11,12,13]. However, the impact of DHC on cell cycle regulation and apoptotic pathways, particularly in cervical cancer, has not been fully elucidated.

Cervical cancer remains one of the leading causes of cancer-related morbidity and mortality among women worldwide [14]. Persistent infection with oncogenic human papillomaviruses (HPVs) disrupts normal cell cycle control, leading to malignant transformation [15]. A key molecular event in cervical carcinogenesis includes the dysregulation of the G1-to-S phase transition, characterized by alterations in cyclin D1, CDKs, and the retinoblastoma (RB) tumor suppressor pathway [16,17]. Cyclin D1 overexpression promotes RB phosphorylation, releasing E2F transcription factors that drive S-phase entry and uncontrolled cellular proliferation—a hallmark of cancer [18]. Therefore, strategies that restore G1 phase arrest and prevent cell cycle progression represent promising therapeutic approaches.

Moreover, cancer cell survival is orchestrated through complex signaling networks: notably, the nuclear factor-κB (NF-κB) and mitogen-activated protein kinase (MAPK) pathways, which can be activated by pro-inflammatory cytokines such as tumor necrosis factor-α (TNF-α) [19]. Although TNF-α can induce apoptosis, it paradoxically activates survival signaling via TAK1, a serine/threonine kinase that plays a central role in propagating both NF-κB and MAPK signaling cascades [20]. TAK1 activation involves complex interactions with TAK1-binding proteins (TAB1, TAB2, TAB3), phosphorylation at key residues, and polyubiquitination, all of which are essential for full kinase activity [21].

The TAK1-mediated activation of the IκB kinase (IKK) complex leads to the phosphorylation and nuclear translocation of NF-κB subunits, promoting the transcription of anti-apoptotic genes such as c-FLIP and A20 [22,23]. Additionally, TNF-α has been shown to activate the epidermal growth factor receptor (EGFR) through ERK- and p38 MAPK-dependent phosphorylation, further enhancing pro-survival signaling and attenuating cell death [24,25]. Thus, TAK1 simultaneously governs two major survival pathways, underscoring its potential as a therapeutic target to sensitize cancer cells to TNF-α-induced apoptosis [20,26].

Given the critical roles of TAK1, NF-κB, and EGFR signaling in promoting tumor cell survival and therapy resistance, a deeper understanding of how DHC modulates these pathways could reveal novel strategies for cervical cancer treatment. Despite promising data on DHC’s pro-apoptotic effects in other cancer types [10,12,13], its influence on TAK1-mediated survival signaling and G1 cell cycle regulation in cervical carcinoma cells has yet to be explored.

Therefore, the primary objective of this study was to investigate whether DHC enhances TNF-α-induced G1 cell cycle arrest and apoptosis in human cervical cancer HeLa cells and to elucidate the underlying molecular mechanisms, particularly focusing on the TAK1-NF-κB and MAPK-EGFR signaling pathways. Our findings provide new insights into the anti-cancer potential of DHC and suggest that it may serve as a valuable adjuvant agent to overcome survival signaling and improve the efficacy of TNF-α-based therapeutic strategies in cervical cancer.

## 2. Results

### 2.1. Treatment of Cancer Cells with TNF-α and Dihydrocapsaicin Decreased Cell Viability

**Figure 2:**
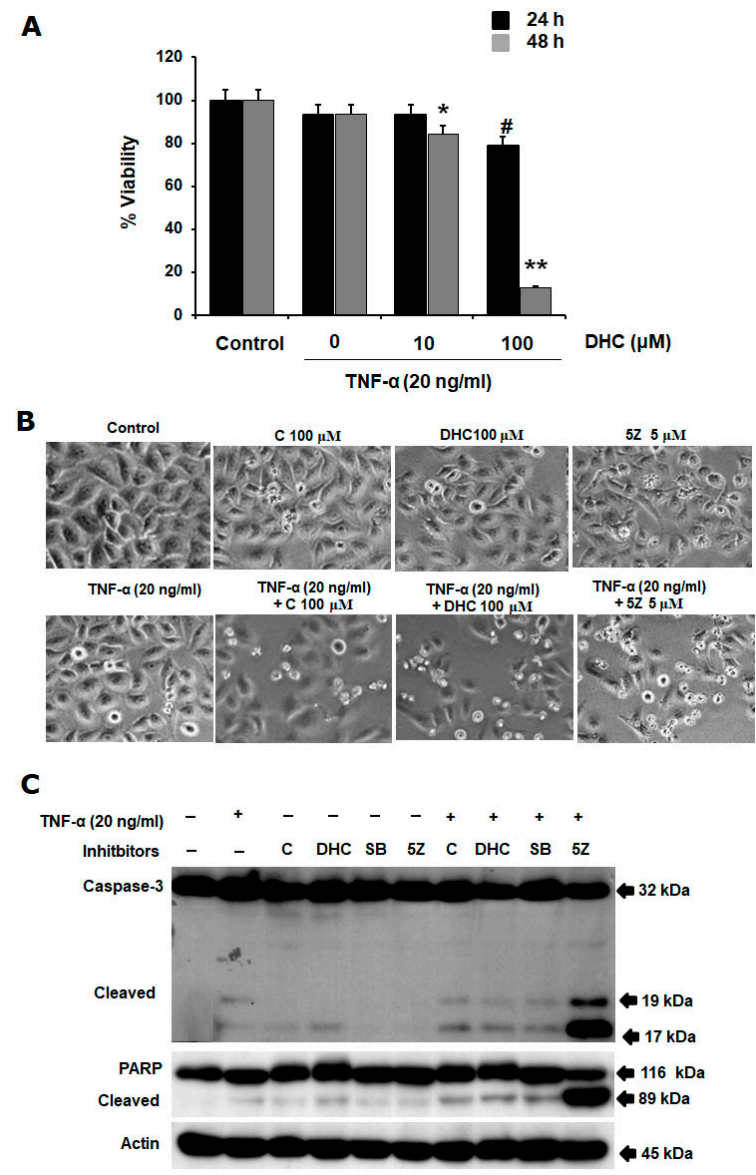
Effects of dihydrocapsaicin on TNF-α-induced apoptosis. (**A**). The viability and (**B**) cell morphology of the HeLa cells were assessed using the WST-1 assay after pretreatment with dihydrocapsaicin (DHC), capsaicin (C), and 5Z with or without TNF-α. (**C**). Prior to being stimulated with TNF-α (20 ng/mL) for 4 h, HeLa cells were pre-treated with DHC, C, SB, and 5Z for 30 min. Anti-caspase-3, PARP and actin antibodies were used with whole-cell extracts prepared, separated, and evaluated by Western blot. Caspase and PARP cleaved forms are shown by the arrows. (* the difference between DHC with and without TNF-α *p* < 0.05, ** the difference between DHC with and without TNF-α *p* < 0.01, # the difference between control and DHC group *p* < 0.05, + with test compound, − without test compound).

To test the effects of DHC on cancer cell viability, HeLa cells were treated with DHC with or without TNF-α 20 ng/mL. 24 and 48 h after the treatment, DHC significantly enhanced TNF-α-induced cancer cell death in a concentration-dependent manner, since the cytotoxicity at 48 h was higher than at 24 h (Figure 2A). Regarding the mode of cell death, we found that DHC enhanced TNF-α-induced cell death via the apoptosis pathway, indicated by cell morphology changes including cells shrinking, becoming smaller, and detaching from the culture surface after cells were treated with 100 µM DHC in the presence of TNF-α for 24 h compared with standard substances, including C, 5Z (5z-7-oxozeaenol, TAK1 inhibitor), and SB (SB 203580, p38 MAPK Inhibitor) (Figure 2B), which correlate with the presence of caspase-3 and poly (ADP-ribose) polymerase (PARP-1) cleavage (Figure 2C).

### 2.2. Effect of Dihydrocapsaicin on TNF-α-Mediated G1 Cell Cycle Arrest at 12 h

**Figure 3:**
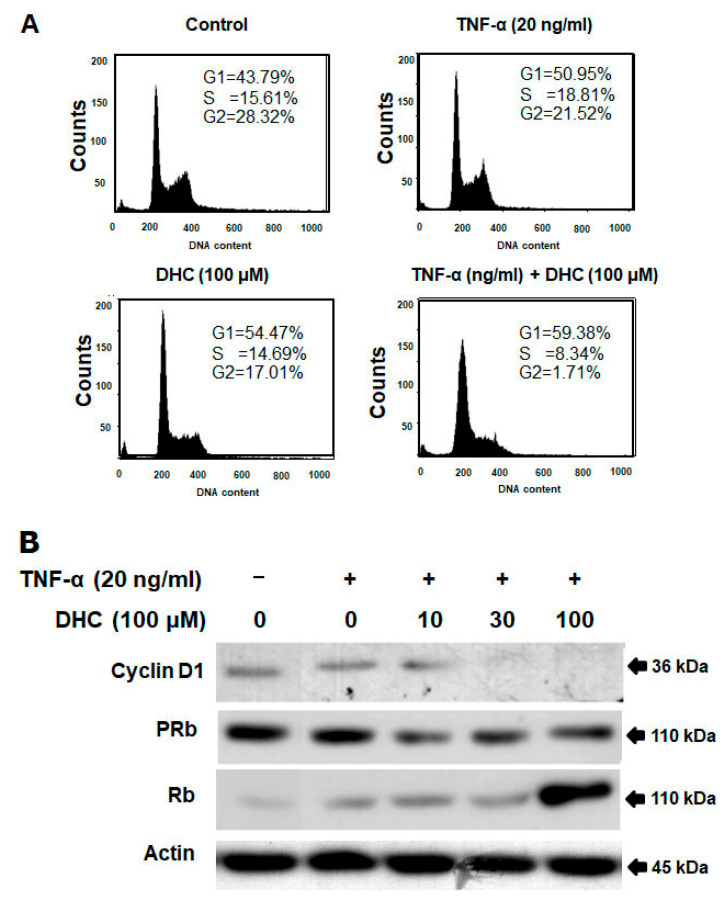
The impact of DHC on cancer cell cycle arrest caused by TNF. (**A**) DHC was pretreated in either the presence or absence of TNF-α 20 ng/mL for 12 h on HeLa cells. Cell cycle analysis was used to examine cell stages. (**B**) For 4 h, cells were exposed to DHC at several doses with or without TNF-α (20 ng/mL) present. Western blotting was used to separate the whole-cell extract and examine it using anti-phospho-RB, cyclin D1, RB, and actin antibodies. (+ with test compound, − without test compound).

The cell cycle may be regulated by DHC-enhanced TNF-α-mediated cell proliferation suppression (Figure 3A). In order to determine the cell cycle location after DHC pre-treatment for 30 min and treatment with or without TNF-α 20 ng/mL for 12 h, the cell cycle phases were analyzed by flow cytometry. The presence of DHC (100 µM) with TNF-α considerably increased the ratio in the G1 phase, which was accompanied by a simultaneous decrease in the proportion of cells in the G2 phase. We examined the impact on the expression of important proteins involved in the regulation of the G1 cell cycle in order to investigate the mechanistic basis of DHC’s growth-inhibiting effects. Co-treatment with DHC and TNF-α, in comparison to the control group, decreased RB phosphorylation, inhibited cyclin D1 expression, and increased RB expression in a dose-dependent manner (Figure 3B). These results indicate that DHC collaborated with TNF-α to regulate cell cycle regulatory proteins, leading to G1 cell cycle arrest. Altogether, these findings demonstrate that DHC has cytostatic properties against cancer cells, especially in the presence of TNF-α, by inducing cell cycle arrest in the G1 phase correlated with the downregulation of cyclin D1 expression and RB protein phosphorylation.

### 2.3. Effect of Dihydrocapsaicin and TNF-α on TAK1/NF-κB Signaling Pathway

**Figure 4:**
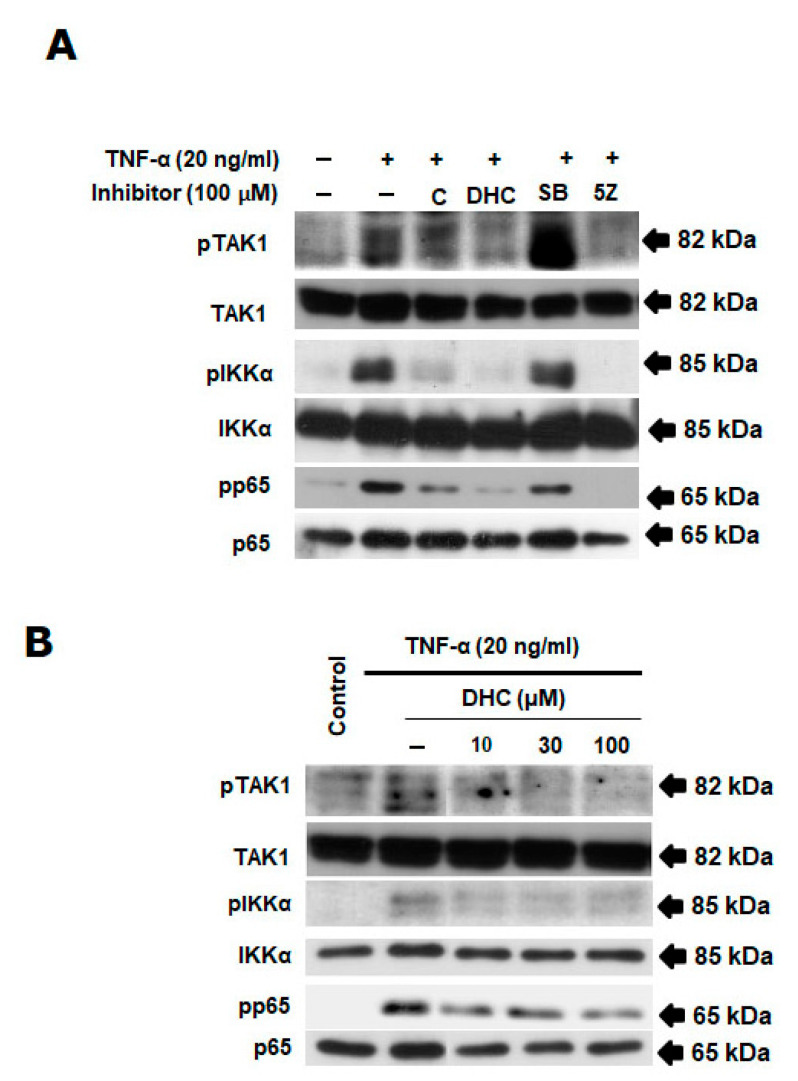
The combination of dihydrocapsaicin and TNF-α inhibited TAK1/NF-κB signaling pathway. HeLa cells were pretreated with dihydrocapsaicin in the specified concentrations for 30 min before TNF-α (20 ng/mL) stimulation for 4 h. The preparation, fractionation, and analysis of a whole-cell extract was performed using Western blotting. (**A**,**B**) Effects of dihydrocapsaicin on TNF-α-induced TAK1/NF-κB activation. Western blotting using anti-pTAK1, TAK1, pIKK-α, IKK-α, pp65, p65, and actin antibodies. (+ with test compound, − without test compound).

The HeLa cells were evaluated for the impact of DHC and C on TNF-α-induced NF-κB activation. Cells were incubated for 30 min with DHC, C, SB, and 5Z before being stimulated with TNF-α for 4 h. The TNF-α-triggered NF-κB pathway’s activation was examined using Western blot. Figure 4A,B shows that DHC inhibited the phosphorylation of TAK1, IKKα, and p65 in a concentration-dependent manner compared with the positive control (inhibitor of TAK1 and p65). These outcomes suggest that DHC inhibited TNF-α-induced TAK1-mediated NF-κB pathway’s activation.

### 2.4. Effect of Dihydrocapsaicin and TNF-α-on EGFR/p38/Erk Signaling Pathway

**Figure 5:**
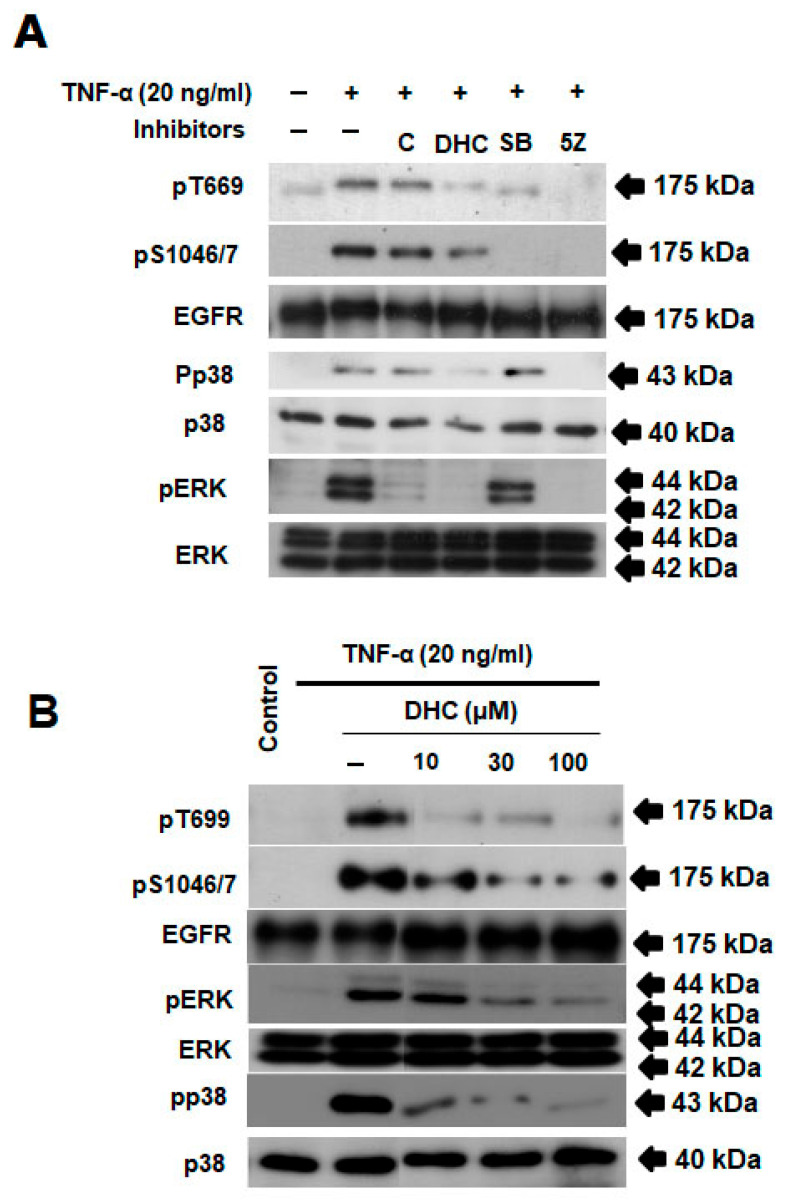
The combination of dihydrocapsaicin and TNF-α inhibited EGFR/p38/Erk signaling pathways. HeLa cells were pretreated with dihydrocapsaicin in the specified concentrations for 30 min before TNF-α (20 ng/mL) stimulation for 4 h. The preparation, fractionation, and investigation of a whole-cell extract was performed using Western blotting method. (**A**,**B**) Effects of dihydrocapsaicin on TNF-α-induced EGFR/p38/Erk activation. Western blotting using anti-p699, TAK1, pS1046/7, EGFR, pErk, Erk, pp38, p38, and actin antibodies. (+ with test compound, − without test compound).

Additionally, we investigated DHC’s impact on the TAK1-mediated phosphorylation of the EGFR at Ser-1046/7 and Thr-669, pathways involved in TNF-α signaling that prevent apoptosis. Figure 5A,B demonstrate that DHC inhibited the phosphorylation of Ser-1046/7 and Thr-669 brought on by TNF-α. We have indicated that the p38 and ERK pathways, respectively, are involved in the phosphorylation of the EGFR at the Ser and Thr residues. This is related to the finding that DHC inhibited the stimulation of p38 and ERK in a concentration-dependent manner when compared to the positive control (inhibitor of TAK1 and p65) (Figure 4A,B).

### 2.5. Effect of Dihydrocapsaicin and TNF-α-on Akt/JNK Signaling Pathway

**Figure 6:**
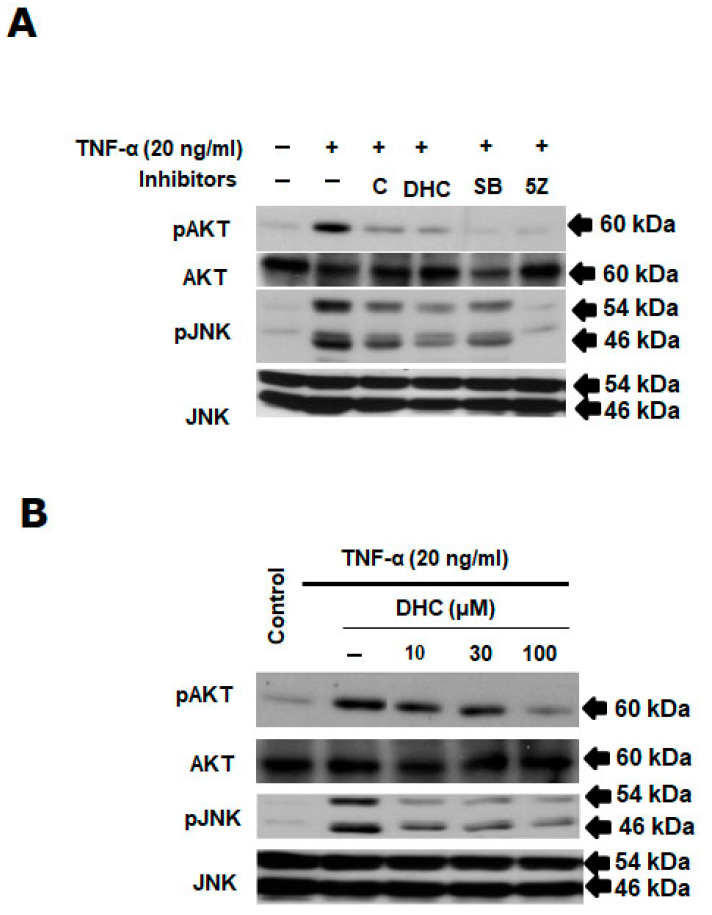
The combination of dihydrocapsaicin and TNF-α inhibited Akt/JNK signaling pathway. Dihydrocapsaicin was pretreated in the specified concentrations for 30 min on HeLa cells before TNF-α (20 ng/mL) stimulation for 4 h. The preparation, fractionation, and investigation of a whole-cell extract was performed using Western blotting method. (**A**,**B**) Effects of dihydrocapsaicin on TNF-α-induced Akt/JNK activation. Western blotting using anti-pAKT, AKT, pJNK, JNK, and actin antibodies. (+ with test compound, − without test compound).

AKT and JNK are a kinase downstream of TAK1 in cancer cells. We used pharmacological inhibitors of TAK1 and p38 to further describe the activation of the TAK1-p38 pathway. When TNF-α-induced phospho-TAK1 was inhibited by the TAK1 inhibitors 5Z-7-oxozeaenol and SB203580, the phosphorylation of its downstream kinases, such as JNK and AKT, also decreased. DHC inhibited the phosphorylation of AKT and JNK in a concentration-dependent manner compared with the positive control (inhibitor of TAK1 and p65) (Figure 6A,B).

## 3. Discussion

In this study, we demonstrated that dihydrocapsaicin (DHC), a major capsaicinoid found in red peppers, enhances tumor necrosis factor-α (TNF-α)-induced G1 cell cycle arrest and apoptosis in HeLa human cervical cancer cells through the suppression of TAK1-mediated NF-κB and EGFR survival pathways. These findings reveal novel mechanistic insights into the potential anticancer properties of DHC, expanding on prior evidence of capsaicinoid-mediated cytotoxicity toward cancer cells.

Previous reports have shown that DHC inhibits the proliferation and metastasis of various cancers through different pathways, including β-catenin suppression and mitochondrial apoptosis induction [11,12,13]. However, its role in modulating cytokine-induced responses in cancer cells had not been fully elucidated. Our findings reveal that DHC not only enhances TNF-α-induced apoptosis but also sensitizes HeLa cells to TNF-α-mediated cell cycle arrest in the G1 phase. Flow cytometry analysis revealed a significant accumulation of cells in the G1 phase after co-treatment, correlating with a reduction in cyclin D1 levels and decreased RB protein phosphorylation. This suggests that DHC can reinforce cell cycle checkpoints, ultimately limiting cancer cell proliferation.

Importantly, TNF-α is known to trigger both pro-apoptotic and pro-survival pathways. The survival responses predominantly involve the activation of TAK1, which subsequently activates downstream effectors including NF-κB, MAPKs, and EGFR [27,28,29]. Our study shows that DHC suppressed TAK1 phosphorylation and significantly inhibited the activation of key downstream kinases, including IKKα, p65, p38 MAPK, JNK, ERK, and Akt, in a concentration-dependent manner. These results suggest that DHC acts upstream at the level of TAK1 to disrupt multiple prosurvival signals that typically counterbalance TNF-α-induced cell death.

Furthermore, DHC also inhibited the TNF-α-induced phosphorylation of EGFR at Ser-1046/1047 and Thr-669, modifications known to enhance EGFR-mediated survival signaling through the p38 MAPK and ERK pathways [30,31]. Since EGFR activation represents a major mechanism through which cancer cells evade apoptosis, our data indicate that DHC’s ability to block EGFR phosphorylation contributes critically to its chemosensitizing effects. This finding aligns with emerging interest in targeting EGFR and related pathways in solid tumors, including cervical cancer.

Another important observation was that DHC induced apoptosis, as evidenced by morphological changes and PARP-1 cleavage, more effectively when combined with TNF-α than as a single agent. PARP cleavage is a hallmark of caspase-mediated apoptosis [32], supporting the conclusion that DHC tips the balance toward cell death when survival pathways are impaired.

Collectively, our results position DHC as a promising candidate for combinatorial cancer therapies aimed at enhancing cytokine-induced apoptosis. By targeting TAK1-mediated survival signaling, DHC not only sensitizes tumor cells to TNF-α but also potentiates cell cycle arrest and apoptotic responses. Given the critical role of TAK1, NF-κB, EGFR and Akt pathways in therapy resistance and tumor progression, agents like DHC that inhibit these pathways could provide a therapeutic advantage, especially in cancers that are refractory to conventional treatments.

Future studies are warranted to investigate the in vivo efficacy of DHC in animal models of cervical cancer, as well as its potential synergistic effects with other chemotherapeutic agents or targeted therapies. Moreover, a deeper exploration into the structural basis of DHC’s interaction with TAK1 and upstream regulators such as TAB1/2/3 could facilitate the design of more potent derivatives with enhanced anticancer activity.

**Figure 7:**
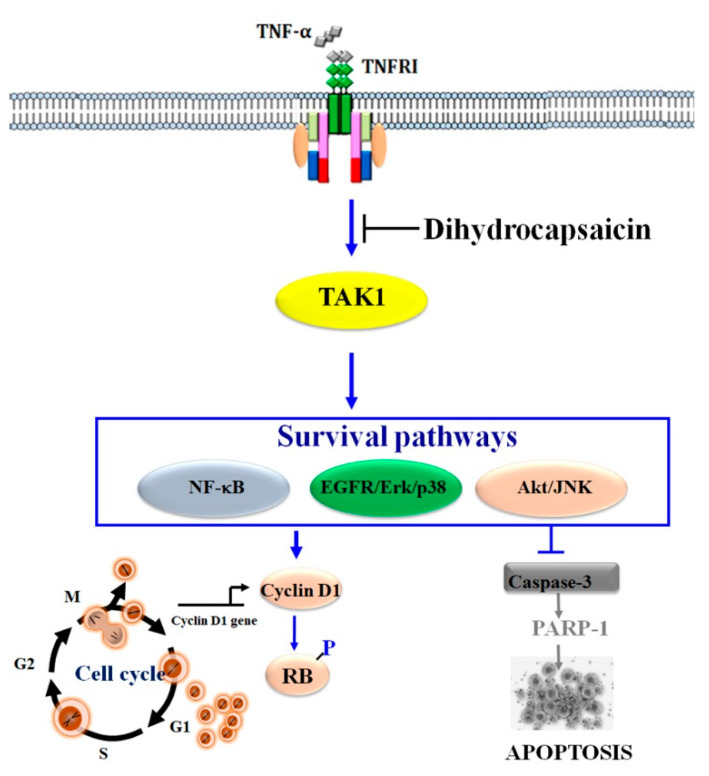
Mechanism of action of dihydrocapsaicin on TNF-α signaling pathways.

In summary, this study expands the understanding of DHC’s anticancer properties and suggests that the modulation of TAK1-mediated survival pathways represents a viable strategy for sensitizing tumor cells to cytokine-induced cell death, particularly in cervical cancer. Our findings support the potential utility of DHC as an adjuvant therapy in the management of malignancies characterized by aberrant NF-κB EGFR and Akt signaling pathways (Figure 7).

## 4. Materials and Methods

### 4.1. Cell Culture and Proliferation Assay

The HeLa cells (ATCC, Manassas, VA, USA) were kept alive by adding 10% fetal calf serum, 100 units/mL penicillin, 100 µg/mL streptomycin, and 5% CO_2_ at 37 °C to Dulbecco’s Modified Eagle Medium (DMEM, high-glucose). WST-1 reagent (4-[3-(4-iodophenyl)-2-(4-nitrophenyl)-2H-5-tetrazolio]-1, 3-benzene disulfonate) was used, and the amount of surviving cells was measured (DOJIN-DO, Kumamoto, Japan). In 96-well microplates with 6 × 10^3^ cells/well, HeLa cells were cultivated before being treated for 24 and 48 h. After adding the dihydrocapsaicin-containing media to the wells, the cells were allowed to stand for 30 min before being stimulated with TNF-α 20 ng/mL. 10 µL of WST-1 was added after 24 h of incubation, and then the absorbance at 450 nm was evaluated.

### 4.2. Cell Cycle Analysis

The Nuclear-ID™ Red cell cycle kit was utilized when the cell cycle was examined (Enzo, Life Science, Farmingdale, NY 11735, USA). The cells were collected, adjusted to have 1 × 10^6^ cells/mL, washed in PBS, and fixed in 70% ethanol. After centrifuging, cells were re-suspended in DNA staining solution and incubated at 37 °C for 30 min. Using flow cytometry, it was possible to determine the proportion of cells and the DNA content in each stage of the cell cycle.

### 4.3. Preparation of Cell Extracts

Dihydrocapsaicin and TNF-α were administered to the cells before whole-cell lysates were made using a lysis buffer (25 mM HEPES pH 7.7, 0.3 mM MgCl2, 0.2 mM EDTA, 10% Triton X-100, 20 mM β-glycerophosphate, 1 mM sodium orthovanadate, 1 mM phenyl-methylsulfonyl flu). After being centrifuged at 14,000 rpm for 10 min, the cell lysate was extracted from the supernatant.

### 4.4. Immunoblotting

SDS-PAGE was used to separate the cell lysate, and it was then transferred to an Im-mobilon-P-nylon membrane (Millipore). BlockAce (Dainippon Pharmaceutical Co., Ltd., Suita, Japan) was incubated with the membrane before primary antibodies—including anti- -phospho TAK1, TAK1, phopho-IKKα (Ser-176/180), IKKα, phospho-p65 (Ser-536), p65, phosphoT669, phosphoS1046/7, EGFR, phospho-p38 (Thr-180/Tyr-182), p38, phospho-Erk (Thr-202, Tyr-204), Erk, phospho-Akt, Akt, caspase-3, PARP, and actin (Cell Signaling Technology, Boston, MA, USA)—were used to identify it. Anti-rabbit, anti-mouse, and anti-goat IgG conjugated with horseradish peroxidase (DAKO, Glostrup, Denmark) were used to detect antibodies, and the enhanced chemiluminescence technique (ECL) was used to see the results (Amersham Biosciences, UK Ltd., Buckinghamshire, UK).

### 4.5. Statistical Analysis

Data was analyzed by using IBM SPSS statistics Version 24. The analysis was conducted employing the one-way analysis of variance (ANOVA) statistic. The mean ± standard deviation was used to express the measurement data. Statistically significant differences were defined to be those with a *p*-value less than 0.05.

## 5. Conclusions

Our study demonstrates that dihydrocapsaicin (DHC) significantly enhances TNF-α-induced G1 cell cycle arrest and apoptosis in HeLa cervical cancer cells by targeting the TAK1-mediated NF-κB and EGFR survival pathways. These findings highlight the therapeutic potential of DHC as a sensitizing agent that disrupts key prosurvival signaling mechanisms commonly activated in cancer. By impairing TAK1 activation and its downstream effectors, DHC not only reinforces the antiproliferative and pro-apoptotic effects of TNF-α but also overcomes cellular resistance mechanisms associated with NF-κB and EGFR signaling. This work provides a compelling rationale for the further investigation of DHC as an adjuvant in combination cancer therapies, particularly for malignancies with hyperactive inflammatory and survival pathways.

## Data Availability

The data generated in the present study may be requested from the corresponding author.
